# First Identification and Genomic Characterization of NADC34-Like PRRSV Strains Isolated from MLV-Vaccinated Pigs in Korea

**DOI:** 10.1155/2023/9995433

**Published:** 2023-04-17

**Authors:** Seung-Chai Kim, Hwan-Ju Kim, Sung-Hyun Moon, Ho-Seong Cho, Won-Il Kim

**Affiliations:** College of Veterinary Medicine, Jeonbuk National University, 79 Gobong-ro, Iksan 54596, Republic of Korea

## Abstract

NADC34-like porcine reproductive and respiratory syndrome viruses (PRRSVs) are emerging globally. Although lineage 1 PRRSVs are emerging in Korea, NADC34-like PRRSV strains have not been detected to date. This study reports the isolation of the first NADC34-like PRRSV strains from samples of MLV-vaccinated animals on a Korean farm with a severe PRRS outbreak causing high mortality and abortion rates in July 2022. Three PRRSV strains with a whole genome length of 15,088 bp were isolated from the outbreaks, and their phylogenetic, recombination properties, and in vitro growth kinetics were evaluated comprehensively. These Korean strains clustered with those of American and Chinese sublineage 1.5 (NADC34-like strains), according to phylogenetic analyses based on the ORF5 gene and the whole genome. All three complete genome sequences originated from recombination between the RespPRRS MLV and NADC34-like strains, according to recombination analysis. Full-length Nsp2 sequence alignment indicated that all three isolates had a continuous deletion in 100 aa, which was similar to that of the IA/2014/NADC34. Based on phylogenetic analysis and the Nsp2 deletion pattern, the isolates are identified as NADC34-like PRRSV. Growth kinetic analysis based on primary porcine alveolar macrophages (PAMs) and MARC-145 cells has revealed that Korean NADC34-like PRRSV strains had better replication ability in primary PAMs than other Korean lineage 1 viruses or the reference strain VR2332, implying higher pathogenicity in pigs. Considering the fact that NADC34-like PRRSV strains were detected in MLV-vaccinated animals on a farm in the area of the highest pig population density in Korea, additional vaccine failure cases and the spread of the virus could be detected in the near future. Collectively, these results aid in comprehending the current epidemic state of PRRSVs in Korea and highlight the need for continuous surveillance.

## 1. Introduction

Porcine reproductive and respiratory syndrome (PRRS) has been one of the most important epidemic diseases affecting the global swine industry since its first recognition in the United States in 1987 [1, 2]. PRRS virus (PRRSV), the causative agent, of the genus *Betaarterivirus* and family *Arteriviridae* of the order *Nidovirales*, is an enveloped, single-stranded positive-sense RNA virus with a genome approximately 15 kb in length that encodes a 5′ untranslated region (UTR), at least 11 open reading frames (ORFs), a 3′-UTR and a 3′-poly(A) tail [3–5]. PRRSVs are classified into PRRSV1 (European type, prototype strain Lelystad virus) and PRRSV2 (North American type, prototype strain VR-2332 virus), which are designated *Betaarterivirus suid 1* and *Betaarterivirus suid 2*, respectively (ICTV2021). The mutation rate of RNA viruses is known to be high due to the lack of 3′ to 5′ exonuclease proofreading ability in RNA-dependent RNA polymerase, and the calculated nucleotide substitution rate of PRRSV is the highest reported for an RNA virus [5–7]. ORF5 gene encodes the major envelop glycoprotein GP5, which is known to be crucial for infectivity, viral assembly, and neutralizing antibodies [8, 9]. Due to its high genetic diversity, ORF5 is commonly used for phylogenetic analysis and lineage classification [10]. In PRRSV2, there are nine lineages (lineage 1 to 9) classified according to ORF5 sequence differences [11, 12].

In South Korea, the field infection of PRRSV2 was first reported in the mid-1980s [13]. Despite the fact that South Korea adopted two PRRSV2 MLV vaccines in 1996 (Ingelvac® PRRS MLV) and 2014 (Fostera® PRRS), PRRSV2 has been widely distributed throughout the country for decades and has formed distinct nation-specific clades referred to as lineage KOR A (LKA), B (LKB), and C (LKC), which are genetically distinct from prevalent common global PRRSV strains and commercial vaccine strains [5, 14, 15]. The majority of Korean PRRSV2 field isolates have been characterized as strains of either Korean lineages or vaccine-variant lineage 5 (L5) [14]. In 2019, however, PRRSV lineage 1 (L1), which is actively circulating in Korea, became the second-largest population (29.6%) of PRRSV strains detected in Korea after its first detection in 2014 [16].

Lineage 1 PRRSV had initially been reported in Canada in the 1990s before spreading across the United States [12, 17], where it has become the most diverse—including sublineages 1.1-1.9—and the most widespread lineage [18]. After the initial outbreak of sublineage 1.9 PRRSV (representative strain: MN184) in North America in the early 2000s [19], sublineage 1.8 (NADC30-like) strains spread from Canada to the United States and China in 2013 [20]. Sublineage 1.5 (NADC34-like) strains started to emerge in the United States in 2014, and have been characterized by severe clinical signs such as high mortality among piglets and abortion “storms” in sow herds within multiple reports [21, 22]. Subsequently, NADC34-like viruses were detected in Peru in 2015 [23] and in China in 2017 [24]. Since 2020, the NADC34-like PRRSVs have rapidly become prevalent in China [17, 25–29]. The PRRSV strains circulating in Korea are mostly NADC30-like strains. Sublineage 1.6 PRRSVs have been detected since 2018 [16]. Although potential recombinant PRRSV strains of NADC30 and NADC34 were identified in a previous study [5], NADC34-like PRRSV strains have never been detected in Korea.

However, during routine PRRSV surveillance conducted by our laboratory and the Jeonbuk National University Veterinary Diagnostic Center (JBNU-VDC), potential NADC34-like PRRSV infection cases have been detected from conventional pig farms in Korea. Therefore, the objective of this study was to genetically characterize by whole genome sequencing and identify the in vitro growth performance of potentially newly emerging NADC34-like PRRSV in Korea.

## 2. Materials and Methods

**Figure 1:**
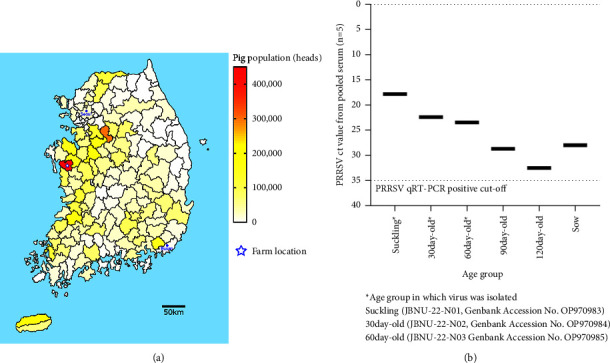
Korean NADC34-like porcine reproductive and respiratory syndrome virus (PRRSV) outbreak information. (a) Geographical distribution of the pig population in Korea with the location of the farm. The farm with the NADC34-like PRRSV outbreak is indicated with a blue star (★). (b) PRRSV quantitative real-time RT‒PCR patterns of pooled serum samples (*n* = 5) for each age group (suckling, 30, 60, 90, 120-day-old, and sow) from the farm. Age groups in which PRRSV isolation was conducted are indicated with asterisk symbols (^*∗*^).

In July 2022, a suspicious PRRS outbreak with high mortality and a high abortion rate was reported on a pig farm with Ingelvac® PRRS MLV-vaccinated animals. The farm is located in Hongseong County, Chungcheongnam-do Province, an area with the highest pig population (442,010 pigs) and the number of farms (198 farms) in Korea (average: 40,084 pigs and 18 farms per administrative division) (KOSIS: Korean Statistical Information Service, https://kosis.kr/eng/) (Figure 1(a)). A total of 30 clinical serum samples from pigs in different age groups (suckling, 30-day-old, 60-day-old, 90-day-old, 120-day-old, and sow) with suspected PRRSV infection were collected and transferred to Jeonbuk National University Veterinary Diagnostic Center (JBNU-VDC). In response to the client's request and the routine protocol for PRRSV diagnostics at JBNU-VDC, five single serum samples from each age group were pooled into one sample representing the age group. Pooled samples were tested with qRT‒PCR with a one-step qRT‒PCR kit after the extraction of viral RNA as described previously [30]. Additionally, the ORF5 gene was amplified from five single pig serum samples within the pooled sample with the lowest Ct value, as previously described.

The complete genome and different genomic parts were compared with reference strains, including NADC34-like PRRSV isolates, using Clustal Omega software [31]. To assess the deletion patterns of nonstructural protein 2 (Nsp2), amino acid (aa) alignments were conducted between the isolates and representative strains using PAL2NAL software [32] as previously described [33]. Maximum-likelihood (ML) phylogenetic trees were constructed based on the complete genome, Nsp2, and ORF5 nucleotide sequences, together with those of the PRRSV2 reference strain and Korean PRRSV2 field isolates. The trees were generated with RAxML-NG software [34] using the GTRGAMMA nucleotide substitution model and 1000 replicates of bootstrap test. The alignment of the complete genome was screened with RDP4 software to check for potential recombination [35]. Potential recombination events were tested by seven different algorithms (RDP, GeneConv, BootScan, MaxChi, Chimeara, SiScan, and 3Seq) with Bonferroni correction [17]. Detection using all seven methods implemented in RDP4 was taken as significant evidence for recombination.

For in vitro growth kinetics evaluation, the JBNU-22-N01 strain, the PRRSV2 reference strain VR2332 (GenBank accession number: AY150564), the Korean NADC30-like strain JB15-N-PJ73-GN (GenBank accession number: MZ287318), and the Korean lineage 1 recombinant strain (NADC34 as major parent and NADC30 as minor parent) [5] JBNU-19-N01 (GenBank accession number: MZ287314) were selected for comparison. All viruses were prepared by propagation and titration in primary PAMs. MARC-145 cells and primary PAMs were infected with each virus at a multiplicity of infection (MOI) of 0.001. Immunofluorescence assays (IFAs) were conducted for infected primary PAMs (24 hours postinfection (hpi)) and MARC-145 cells (48 hpi) as previously described [36]. Supernatants were collected at 0, 24, 48, 72, and 96 hpi, and the virus in these supernatants was titrated by the cytopathic effect (CPE) expressed as the 50% tissue culture infective dose (TCID50) according to the Reed–Muench method by infecting the same cell type in which viruses were propagated.

## 3. Results and Discussion

The levels of PRRSV viral RNA in pooled serum samples were highest in the suckling piglet age group (Ct value 17.89), followed by weaners (30-day-old, Ct value 22.43), growing pigs (60-day-old, Ct value 23.58), sows (Ct value 28.05), and fattener groups (90-day-old, Ct value 28.69, and 120-day-old, Ct value 32.46), a pattern suggesting that maternal antibodies transferred from sows to piglets do not offer protective immunity, resulting in vertical transmission in the herd (Figure 1(b)). In the subsequent ORF5 phylogenetic analysis after sequencing the ORF5 gene directly from clinical serum samples, the PRRSV strain circulating in the farm was genetically distinct (>12% nucleotide distance) from all other lineages previously identified in Korea as well as the Ingelvac® PRRS MLV that was used at the farm before the outbreak (data not shown).

Three PRRSV strains, JBNU-22-N01 (GenBank accession number: OP970983), JBNU-22-N02 (OP970984), and JBNU-22-N03 (OP970895), were successfully isolated from each age group (suckling, 30-day-old, 60-day-old and 90-day-old piglets) by propagation in primary PAMs. Complete genomes of each virus were successfully gathered through the NGS pipeline. The genomes of JBNU-22-N01, JBNU-22-N02, and JBNU-22-N03 shared >99.9% identity with each other. Genome alignments revealed that the Korean NADC34-like PRRSVs shared 82.4% identity with RespPRRS MLV (Ingelvac® PRRS MLV, L5), 85.1% identity with NADC30, 84.3% identity with ISU30, and 90.1% identity with NADC34 (Table 1). The nucleotide homology of ORFs 2–7 between the newly isolated PRRSVs and NADC34 was 96.0%, which was greater than the homology shared with other typical strains. ORFs 2–7 of the newly isolated PRRSVs shared 96.0% nucleotide homology with NADC34, which was higher than the homology shared with other representative strains. However, ORF1a and ORF1b shared 86.5% and 90.7% nucleotide homology with NADC34, which is a relatively lower homology than ORFs 2–7. Among the 16 nonstructural proteins encoded by ORF1a and ORF1b, some of the Nsps shared the highest nucleotide and aa homology with representative strains other than NADC34, such as Nsp3 (92.4% nucleotide and 96.5% aa homology with NADC30); Nsp4-6 (91.3–97.8% nucleotide and 93.4–100% aa homology with RespPRRS MLV); and Nsp8, Nsp11, and Nsp12 (90.3–94.6% nucleotide and 93.1–98.7% aa homology with ISU30) (Table 1).

**Figure 2:**
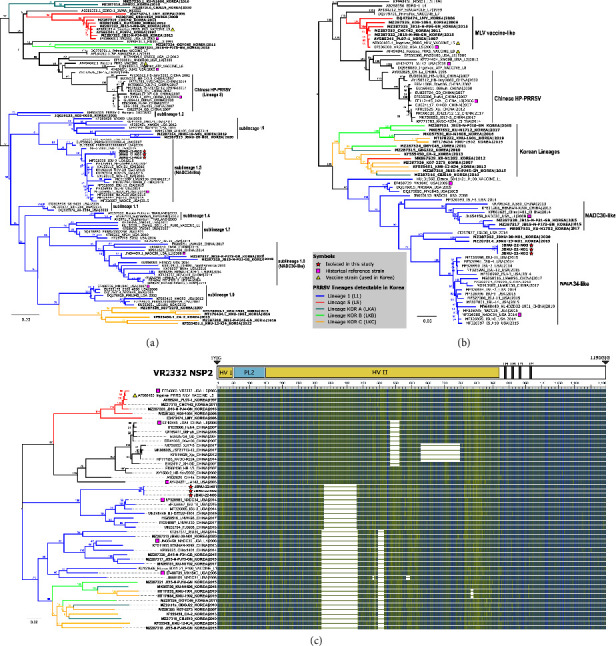
Phylogenetic analysis of NADC34-like porcine reproductive and respiratory syndrome virus (PRRSV) strains and identification of amino acid deletion patterns in Nsp2. (a) Maximum-likelihood (ML) phylogenetic tree constructed based on the ORF5 gene of the novel NADC34-like PRRSV isolates and reference PRRSV strains from each lineage. (b) ML phylogenetic tree constructed based on full-length genomes of the novel NADC34-like PRRSV isolates and reference PRRSV strains from each lineage. (c) Schematic diagram of multiple alignments of Nsp2 amino acid sequences. The tree on the left was generated based on NSP2 nucleotide sequences, and multiple sequence alignments (MSAs) were generated by using the NCBI MSA tool version 1.22.1 with coloration based on the BLOSUM62 matrix. Lineages of PRRSV2 are color-coded in the trees and include lineages 1 (blue), 5 (red), KOR A (teal), KOR B (lime), and KOR C (orange). Historical reference strains, including VR2332, JA142, JXA1, MN184, NADC30, and NADC34, are labeled with pink squares (■). Vaccine strains are labeled with yellow triangles (▲). Newly obtained Korean NADC34-like sequences are labeled with red stars (★).

Phylogenetic trees were constructed using the ML method to understand the evolutionary relationship between the newly isolated PRRSV strains and the representative strains. Both ORF5-based and complete genome-based phylogenetic trees showed that JBNU-22-N01, JBNU-22-N02, and JBNU-22-N03 clustered with the NADC34-like (sublineage 1.5) cluster (Figures 2(a) and 2(b)). Genomic insertion and deletion can be easily identified among PRRSVs, especially in Nsp2, the largest PRRSV protein, which tolerates aa deletions and insertions of foreign genes [37]. Additionally, Nsp2 has also been reported to be a molecular marker based on certain deletion patterns in various strains, for instance, a 100-aa continuous deletion in Nsp2 of NADC34-like PRRSV [22], a 131-aa discontinuous deletion in Nsp2 of MN184- or NADC30-like PRRSV [38], and a 30-aa discontinuous deletion in Nsp2 of the highly pathogenic PRRSV strain in China [39]. Complete Nsp2 aa sequence alignment reveals that the JBNU-22-N01, JBNU-22-N02, and JBNU-22-N03 strains harbor a 100-aa continuous deletion at residues 328–427 of IA/2014/NADC34 Nsp2, which is consistent with the pattern for NADC34-like PRRSV (Figure 2(c)). Considering the results of phylogenetic analysis and Nsp2 deletion patterns, the three PRRSV isolates were confirmed to be NADC34-like strains, the first to be identified in Korea.

**Figure 3:**
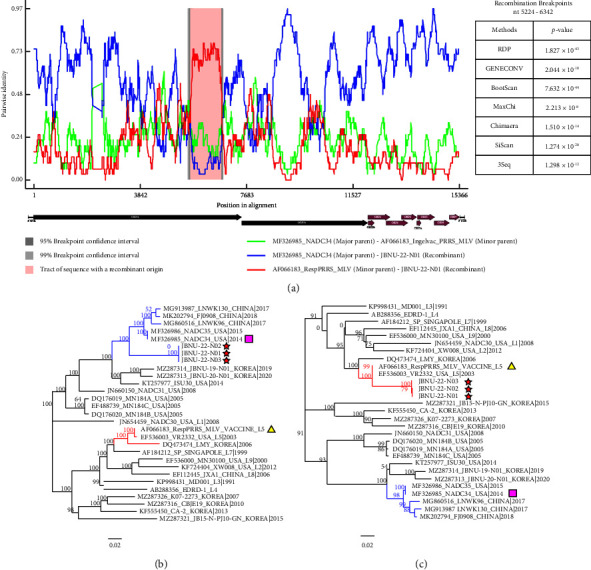
Characterization of the supported recombination events between JBNU-22-N01 and representative PRRSV lineages. (a) Similarity plot and Bootscan analysis of JBNU-22-N01. The *y*-axis indicates the pairwise identity between the major parent and minor parent (lime), between the major parent and the recombinant strain (blue), or between the minor parent and the recombinant strain (red). The range of recombination is shaded in red. (b) Phylogenetic trees based on major parental regions (nt 1–5,523 and nt 6,325−15,088) and (c) the minor parental region (nt 5,524−6,324).

Recombination is a common event in PRRSV isolates and is an advantageous strategy for securing the viral genetic diversity [17, 40]. This leads to the development of new PRRSV genotypes and also may correlate with an increase in virulence [41]. To identify recombination in Korean NADC34-like strains, RDP4 software was used to assess potential recombination events. Recombination events were observed in all three strains, with recombination breakpoints located in Nsp4 (nt 5,524) and Nsp7*α* (nt 6,342) (Figure 3(a)). Phylogenetic trees about each sequence region separated by the breakpoints were generated in order to further verify the potential recombination event [42]. The regions outside the breakpoints (nt 1–5,523 and nt 6,325−15,088) are closely related to the NADC34 strain (Figure 3(b)), and the region between the breakpoints (nt 5,524−6,324) is closely related to the RespPRRS MLV strain (Figure 3(c)). Taken together, the results as noted indicated that the three NADC34-like strains have arisen via recombination between NADC34 and RespPRRS MLV. In fact, considering that the major circulating PRRSV lineage in Korea is MLV-related lineage 5 and that Ingelvac® PRRS MLV has been the most widely used vaccine in the field since its first commercial launch in Korea in 1996 [16], recombination events between circulating PRRSV lineages and RespPRRS MLV have been reported as the most frequent recombination pattern in Korea [5]. At the farm where the NADC34-like strains were isolated, the Ingelvac® PRRS MLV vaccine was administered prior to the outbreak, suggesting that the recombination identified in this study is another case of MLV-field strain recombination. Recently, recombination between local strains and NADC34-like strains as the main parent has been reported in China [17] and the United States [4]. It is necessary to investigate changes in pathogenicity in recombinants that are combined with the MLV vaccine strain and NADC34-like strains in further study.

**Figure 4:**
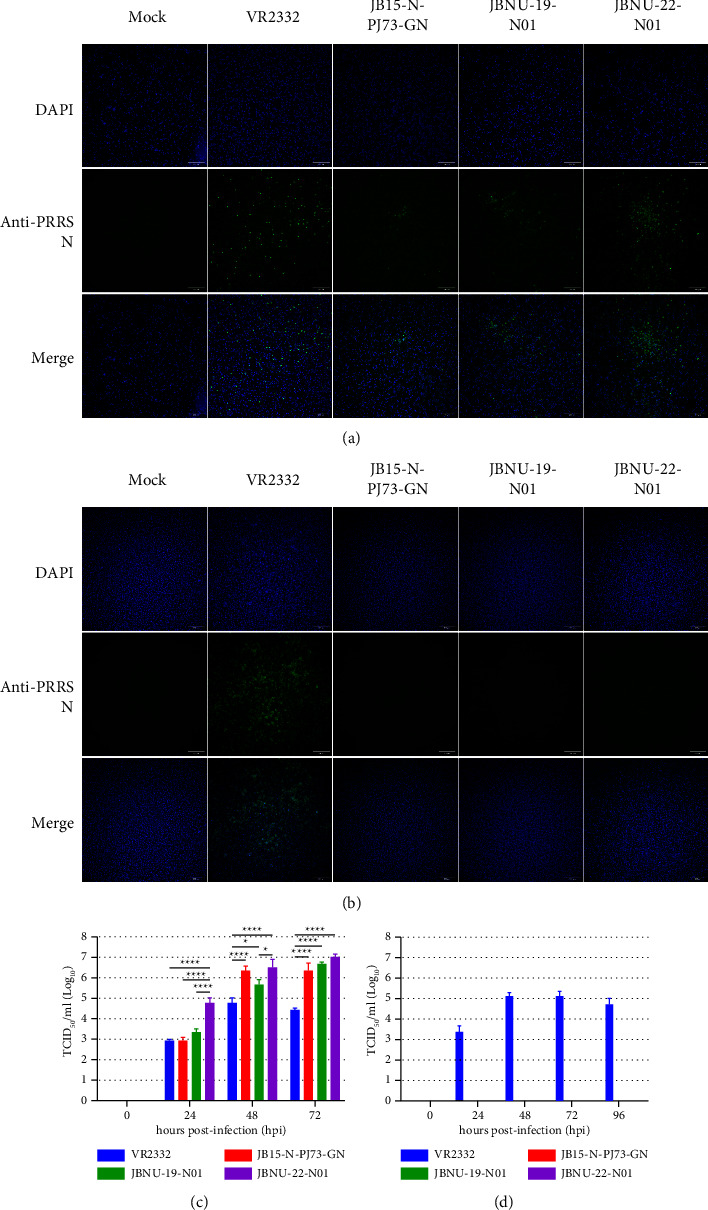
In vitro PRRSV growth kinetics. Indirect immunofluorescence assays (IFA) stained with mouse anti-PRRS N primary antibody and goat anti-mouse IgG H + L FITC secondary antibody followed by DAPI staining for (a) primary PAM culture at 24 hours postinfection (hpi) and (b) MARC-145 cell culture at 48 hpi. Magnification: ×100. Multistep growth curve results of each PRRSV strain in (c) primary PAMs and (d) MARC-145 cells.

PRRSV infection shows a strongly restricted tropism for host species and target cells [36]. PAMs are the PRRSV primary target cells in vivo, and the immortalized African green monkey kidney epithelial cells MARC-145 support viral infection in vitro [43]. Although the MARC-145 cell line has been used as a key platform for PRRSV isolation and vaccine development through large-scale production of PRRSV [36, 44], PRRSV has evolved into different lineages, and many PRRSV strains fail to infect MARC-145 cells [36, 45–47]. According to a recent report, cells expressing porcine CD163, which is the main receptor for PRRSV infection, were susceptible to different PRRSV2 infections, including infection with NADC30-like and NADC-34-like PRRSV strains, which do not replicate in MARC-145 cells [36]. Consistently, the PRRSV2 reference strain VR2332 and Korean lineage 1 viruses (NADC30-like, recombinant lineage 1, and NADC34-like PRRSV strains) successfully replicated in primary PAM culture (Figures 4(a) and 4(c)), while lineage 1 viruses did not replicate in MARC-145 cells (Figures 4(b) and 4(d)), as evidenced by immunofluorescence assays. This result further supports the use of porcine CD163-expressing cells instead of MARC-145 cells for diagnostic investigation as well as autogenous vaccine production [36].

As for PRRSV growth kinetics results in primary PAMs, the virus titers of all Korean lineage 1 PRRSVs were significantly higher than those of VR2332 from 48 to 72 hpi (Figure 4(c)). Interestingly, the titer of the Korean NADC34-like strain JBNU-22-N01 was significantly higher than that of all other viruses at 24 hpi (Figure 4(c)), and the PRRSV-specific CPE in JBNU-22-N01-inoculated PAMs was more severe, with more PAMs falling off the bottom of the cell culture plate (data not shown). A previous report suggested that PRRSV's replication abilities and adaptation to its target cells directly contribute to PRRSV pathogenicity and virulence in attenuated PRRSV in vivo and in vitro reversion to virulence studies [48]. As various strains of NADC34-like strains were reported, pathogenicity was also reported to be diverse, as the American NADC34 strain reveals high pathogenicity [17, 22], while the Chinese PRSV-ZDXYL-China-2018-1 and HLDJZD32–1901 strains have moderate and low pathogenicity, respectively [17, 49, 50]. However, considering the high mortality and abortion rates observed on the farm from which Korean NADC34-like strains were isolated together with the replication ability of PRRSV shown in the in vitro experimental results, it could be implied that the Korean NADC34-like strains show the capability of inducing high pathogenicity in pigs. In vivo experiments to assess the pathogenicity of the Korean NADC34-like strains will be investigated.

The emergence and spread of lineage 1 PRRSVs, including NADC34-like viruses, continue to be reported globally. In Peru, phylogenetic analysis of field samples obtained between 2015–2017 showed that 15 of 20 isolates (75%) were associated with NADC34-like (PRRSV 1-7-4) strains [23]. In China, sublineage 1.5 and sublineage 1.8 strains comprised 28.6% and 35.4%, respectively, of positive samples in 2021, that is, lineage 1 strains were much more predominant than the proportion of other lineages [17]. In Korea, lineage 1 PRRSV comprised the second-largest population (29.6%) of PRRSVs in 2019 [16]. At present, no vaccine for lineage 1 strains is available on the global market [17], and there was only partial protection against lineage 1 PRRSVs in research for five MLVs that were commercially available [51]. In this study, NADC34-like PRRSV-infected animals showed signs of vertical transmission and long-term infection with patterns of the lowest PRRSV Ct value in suckling piglets, an age-wise gradual increase in Ct value, and a relatively low Ct value in sows (Figure 1(b)). Considering that MLV vaccination occurred on the farm prior to the outbreak, it is suggested that the vaccine failed to confer sufficient cross-protection against NADC34-like PRRSV infection, implicating a potential vaccine failure case. Since the outbreak was detected on a farm located in the area with the highest pig population density in Korea (Figure 1(a)), there is an imminent threat of potential NADC34-like PRRSV outbreaks across the Korean swine industry, as most pig farms have no vaccination option other than the current MLV vaccine. Therefore, enhanced biosecurity should be carried out, and increased surveillance is necessary.

## Figures and Tables

**Table 1:** Detailed comparison of the full-length genomes of the Korean NADC34-like strain JBNU-22-N01 and other PRRSV reference strains.

	RespPRRS MLV (%)	NADC30 (%)	ISU30 (%)	NADC34 (%)
*Nucleotides*				
Complete genome	82.4	85.1	84.3	**90.1**
ORF1a	79.2	81.2	80.3	**86.5**
ORF1b	84.4	88.2	**91.2**	90.7
ORF2-7	85.3	88.1	86.8	**96.0**

*Nucleotides/amino acids*				
nsp1*α*	85.7/94.9	89.0/93.1	**92.3/94.9**	**92.7/94.3**
nsp1*β*	75.5/75.2	78.6/76.7	85.5/87.9	**89.2/89.6**
nsp2	71.3/65.5	76.7/72.6	74.2/70.5	**86.6/85.6**
nsp3	82.8/94.2	**92.4/96.5**	77.2/88.1	85.5/92.8
nsp4	**91.3/93.4**	81.8/92.4	80.0/91.2	78.7/90.8
nsp5	**95.1/94.6**	87.4/91.8	83.8/92.0	82.7/89.5
nsp6	**97.8/100**	90.8/100	88.2/100	88.2/93.5
nsp7*α*	78.2/91.9	77.5/91.1	85.9/95.2	**86.6/95.2**
nsp7*β*	86.2/82.1	87.2/75.4	88.0/87.4	**88.7/92.4**
nsp8	86.8/85.7	86.6/88.2	**94.6/93.1**	93.7/93.1
nsp9	85.6/96.0	88.2/96.7	91.6/97.8	**91.7/97.9**
nsp10	83.8/94.9	92.0/98.4	90.2/97.7	**88.9/97.9**
nsp11	83.5/94.0	84.0/94.0	**90.3/94.5**	**90.7/94.0**
nsp12	82.0/90.4	81.9/89	**93.5/98.7**	91.2/96.7
ORF2	87.4/93.5	83.5/82.1	83.6/83.0	**96.2/96.0**
ORF3	81.8/81.9	83.6/82.9	82.3/83.3	**94.8/95.6**
ORF4	84.7/86.2	93.7/95.4	93.0/92.4	**95.9/97.2**
ORF5	85.6/84.3	87.7/91.7	85.4/90.6	**96.6/97.5**
M	86.8/91.6	93/92.2	91.6/91.6	**97.9/98.3**
N	87.3/91.5	93.6/97.5	91.4/94.1	**95.2/96.7**

Bold values indicate PRRSV reference strains with the highest identity of nucleotide and/or amino acid sequence compared to JBNU-22-N01 strain in this study.

## Data Availability

The data that support the findings of this study are available from the corresponding author upon reasonable request.
